# Thyrotoxic Periodic Paralysis With Graves' Disease: A Case Report

**DOI:** 10.7759/cureus.73223

**Published:** 2024-11-07

**Authors:** Abin Thomas, Bilha Baby, Selin C Joy, Sreekrishnan T P, Gireesh Kumar K P

**Affiliations:** 1 Emergency Medicine, Amrita Institute of Medical Sciences, Ernakulam, IND; 2 Department of Pharmacy Practice, Amrita School of Pharmacy, Kochi, IND; 3 Emergency Medicine, Amrita Institute of Medical Sciences, Kochi, IND

**Keywords:** grave’s disease, hyperthyroidism, hypokalemia, thyroid, thyrotoxic periodic paralysis

## Abstract

One type of hypokalemic periodic paralysis that is associated with hyperthyroidism is called thyrotoxic periodic paralysis (TPP). TPP can be linked to any cause of hyperthyroidism, although Graves' disease is the most common cause. This sporadic variant of hypokalaemic periodic paralysis, thyrotoxic periodic paralysis, is characterized by rapid onset weakness in the proximal muscles. Due to their similar presentations, it is sometimes mistaken for familial periodic paralysis (FPP); however, biochemical tests and the presence of thyrotoxic characteristics can help distinguish the two conditions. Treatment options for this reversible illness include rapid potassium replacement and thyroid hormone normalization. To mitigate this potentially fatal complication, knowledge regarding the identification, prompt treatment, and avoidance of future episodes of TPP is crucial.

## Introduction

Hyperthyroidism can lead to an uncommon but serious condition called thyrotoxic periodic paralysis (TPP) [1]. Asian males in their third to fifth decades of life are more likely to have TPP. Hypokalemia coupled with paralysis, which appears as lower limb weakness, is the main characteristic of TPP [2]. However, TPP appears to be declining less often in the Asian population; according to Karndumri et al., the prevalence of TPP in Japanese patients fell by more than 40% between 1957 and 1991 [3]. A hypothesis was put up suggesting that the consumption of more potassium and less carbohydrates by the Japanese populace could be the reason for the decline. Hypokalemic periodic paralysis is characterized by three primary symptoms: hyperthyroidism, muscle paralysis, and transient hypokalemia without a complete potassium deficit in the body [4].

Common causes include a high glucose load or rest following intense activity. Additional precipitants include menstruation, steroid medication, alcohol usage, exposure to the cold, coexisting diseases, and use of beta-agonist bronchodilators [5].

While the exact cause of hypokalemic periodic paralysis is uncertain, it is known that thyroid hormone and hyperadrenergic activity both directly and indirectly increase the activity of the sodium-potassium adenosine triphosphate pump (Na^+^-K^+^ ATPase). The thyroid hormone differentially controls the isoforms of Na^+^-K^+^ ATPase and increases its activity in sensitive tissues. For each adenosine triphosphate (ATP) that is spent, the Na^+^-K^+^ ATPase pumps two potassium (K^+^) into the cell and three sodium (Na^+^) out of the cell. The gradient of higher internal potassium levels and higher external sodium concentrations is maintained by the Na^+^-K^+^ ATPase pump. The excess thyroid hormone in hyperparathyroidism causes an increase in the production of Na^+^-K^+^ ATPase. Moreover, potassium becomes retained in cells due to reduced potassium outflow. As a result, a vicious cycle of sodium channel inactivation and hypokalemia-induced paradoxical depolarization results in muscle inexcitability and paralysis [4].

The most prevalent kind of hypokalemia is caused by hyperadrenergic activity, hyperinsulinemia, and thyroid hormone, which all drive high Na^+^-K^+^ ATPase activity in the pathophysiology of TPP [6].

The Na^+^-K^+^ ATPase pump, which improves the active transport of K^+^ ions into the intracellular compartment, is the cause of hypokalemia in the absence of a potassium deficiency in the entire body. This comes after hyperthyroidism [7]. The increased catecholamine levels and hypokalemia brought on by thyroid hormones conclude that TPP arises from hyperthyroidism. This change is mostly responsible for muscular paralysis [8]. The weakening of muscles in TPP may also be caused by mutations in the Cav1.1 voltage-gated calcium (Ca^+^) channel or Nav 1.4 Na^+^ channel skeletal muscles [9].

Graves' disease is the most common cause of hyperthyroidism associated with TTP. Less frequently occurring causes of thyrotoxicosis include amiodarone- and iodine-induced thyrotoxicosis, lymphocytic thyroiditis, toxic multinodular goiter, excessive thyroxine usage, and solitary toxic adenoma [10].

## Case presentation

**Figure 1:**
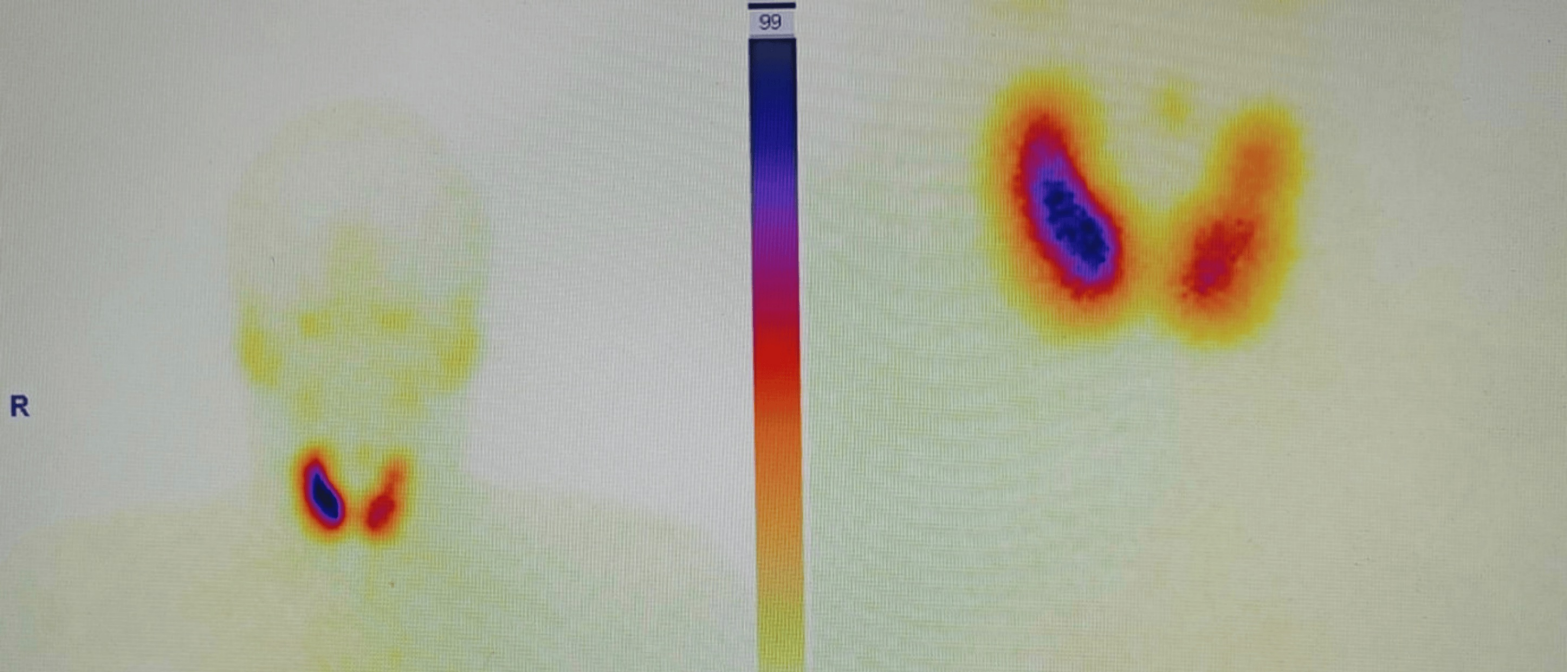
Thyroid scintigraphy report

A 30-year-old male patient with no known concomitant conditions was brought to the emergency department with a one-day history of abrupt onset paralysis in both upper and lower limbs, along with fever. The patient was taking antipyretics for fever. The next morning, he began to have trouble getting upstairs and getting items off of a rack. Fasciculations, fluctuations, and diurnal changes were not present in his past. Upon evaluation, the vital parameters of the patient were all within normal ranges. A neurological test revealed reduced reflexes and a power of 3/5 in all four limbs. It was determined that there was no involvement of the cranial nerve, sphincter, extraocular muscle, or muscular wasting. His laboratory examination showed a potassium level of 1.5mEq/L. High QRS voltage, ST depression, and U waves were seen on the ECG. Blood gas analysis revealed no acid-base disturbance, and the hypokalemia workup revealed indications of non-renal potassium loss. The thyroid function tests in the patient revealed elevated T3 and T4 levels together with low thyroid-stimulating hormone (TSH). Thyroid scintigraphy demonstrated increased thyroid gland entrapment typical of Grave's disease, and an ultrasound of the neck revealed characteristics suggestive of thyroiditis (Figure 1). 

Our ultimate diagnosis was thus thyrotoxic periodic paralysis triggered by infection. Intravenous potassium correction and antithyroid drugs (NeoMercazole® 10 mg tablet thrice daily and propranolol 40 mg tablet twice daily) were initiated for the patient. Furthermore, supportive care and empirical antibiotics were started at the same time. The power in all four limbs recovered to normal, and the serum potassium levels steadily stabilized at 4.3 mEq/L. Results for each laboratory parameter are displayed in Table 1.

**Table 1:** Laboratory parameters

Laboratory investigations	Values obtained	Normal range
Total white blood cells (WBC) count	13000 cell/mm^3^	4500-11000 cells/mm^3^
C-reactive protein (CRP)	30mg/dL	<0.3mg/dL
Serum potassium	1.5 mEq/L	3.5-5.5 mEq/L
Serum magnesium	2.1mg/dL	1.7-2.2 mg/dL
Urine potassium	4 mmol/L	>10 mmol/L
Thyroid-stimulating hormone (TSH)	0.005 u IU/ml	0.4-5.5 u IU/ml
Free T3	6.40 pg/ml	2.0-4.4 pg/ml
Free T4	3.88 ng/ml	1.0-1.6 ng/ml

After four days of stay in the hospital, the patient was discharged with improved symptoms and hemodynamic stability.

## Discussion

A rare manifestation of thyrotoxic periodic paralysis with Graves' disease was presented in our case. One peculiar sequela of hyperthyroidism is thyrotoxic periodic paralysis [11]. Basic muscular symptoms are typical, ranging from paresis to complete paralysis, and are often associated with low blood potassium levels (<3 mmol/l) [8]. The patient had a previous history of fever, whereas lower limb weakness was presented one day prior to admission. The patient was diagnosed with thyrotoxic periodic paralysis with Graves' disease following pertinent testing. He was subsequently diagnosed with TPP associated with Graves' disease. An intravenous potassium correction was administered to treat hypokalemia. Additionally, the patient was initiated NeoMercazole® 10 mg tablet thrice daily, an antithyroid drug. A comparable case with a 40-year-old Asian male patient who arrived with palpitations, diffuse body aches, and sudden onset proximal limb weakness was described by Zahir Hussain et al. [12]. The ECG changes confirmed severe hypokalemia. A similar therapeutic plan, consisting of intravenous potassium correction, propranolol (20 mg, three times a day), and carbimazole (40 mg, daily), was employed in this patient compared to our case.

In a 10-year review conducted by Chang et al. among patients with TPP, 34% had identified a precipitating cause [13]. In the case that we describe, the only potential identified cause was thyrotoxicosis. Adult males between the ages of 20 and 40 are more likely to develop TPP than women between the ages of 40 and 60, who usually have Graves' disease, according to Hussain et al. [14]. A possible explanation for the male preponderance might be the impact of androgens on Na^+^-K^+^ ATPase activity [15]. The management strategy for TPP includes short-term beta-blocker usage, hypokalemia correction, and the use of antithyroid medications [16]. Since carbohydrate consumption is a risk factor for Graves' disease in patients with TPP, patients should be advised to minimize their intake [10].

## Conclusions

In patients presenting with hypokalemic paralysis, thyroid function tests are an important part of assessment. In thyrotoxic patients, imaging of the thyroid may be indicated. As a result of the good management of this case, the patient was discharged in a physically and hemodynamically stable condition. This case provides clinically beneficial information and can be useful in daily clinical practice.
